# Recurrent Pneumothorax in an Adult Male With Bilateral COVID-19 Pneumonia

**DOI:** 10.7759/cureus.17025

**Published:** 2021-08-09

**Authors:** Ayusha Poudel, Anurag Adhikari, Barun B Aryal, Yashasa Poudel, Ishu Shrestha

**Affiliations:** 1 Intensive Care Unit, Nepal Korea Friendship Municipality Hospital, Madhyapur Thimi, NPL; 2 Emergency Medicine, BP Smriti Hospital, Kathmandu, NPL; 3 Anesthesiology and Critical Care, B & B Hospital Pvt. Ltd., Kathmandu, NPL; 4 Internal Medicine, Dhulikhel Hospital, Dhulikhel, NPL

**Keywords:** covid-19, pneumonia, pneumothorax, pleurodesis, recurrent pneumothorax

## Abstract

Pneumothorax is defined as the condition in which air is collected between the visceral and parietal pleura. Pneumothorax as a complication of coronavirus disease 2019 (COVID-19) infection has been reported in relatively few cases and recurrent pneumothorax is even rarer. We present a case of a 50-year-old critically ill patient who required mechanical ventilation for 55 days and developed recurrent bilateral pneumothorax. The patient initially presented with shortness of breath and cough. He was found to be COVID-19 positive on the polymerase chain reaction (PCR) test. Subsequently, his oxygen demand increased, and he ultimately needed mechanical ventilation. He developed four episodes of pneumothorax. The patient was managed in all four episodes with intercostal tube insertion. To prevent subsequent episodes, pleurodesis was performed after the fourth episode of pneumothorax.

## Introduction

The coronavirus disease 2019 (COVID-19) caused by the severe acute respiratory syndrome coronavirus-2 (SARS CoV-2) has a multitude of pulmonary and extra-pulmonary manifestations [1]. However, pneumothorax as a complication of COVID-19 infection has been reported in relatively few cases, with 1% incidence in hospital admitted patients and 2% in those needing intensive care [2]. 

This complication has been seen in critically ill cases of acute COVID-19 infection placed under mechanical ventilation and those receiving oxygen supplementation through a high flow nasal cannula. The possible explanations are spontaneous rupture of virus-infected fragile airways or barotrauma from the various modalities of oxygen supplementation [3]. We present a case of a 50-year-old male with recurrent spontaneous pneumothorax placed on mechanical ventilation for 55 days. The first three episodes developed while on mechanical ventilation and the final episode while receiving oxygen supplementation via nasal cannula. This case is the first of its kind to be reported from Nepal.

## Case presentation

A 50-year-old male presented to the emergency room (ER) with complaints of shortness of breath and dry cough. His known co-morbidities were diabetes mellitus and hypertension for which he was under oral metformin and metoprolol. He was initially managed in the ER where the significant laboratory findings were ketonuria with a random blood sugar level of 432 milligram/deciliter (mg/dL) and COVID-19 polymerase chain reaction (PCR) positive status. He was admitted to the COVID-19 ward and managed with insulin infusion and normal saline. He required oxygen supplementation at 12 liters per minute (L/min) via a face mask with a reservoir bag. The peripheral oxygen saturation was maintained at 94%. He was started on IV piperacillin/ tazobactam 4.5 g thrice daily, IV remdesivir 200 mg initial dose followed by 100 mg daily for five days, IV dexamethasone 6 mg twice daily, and subcutaneous enoxaparin 40 mg once daily. The ketonuria resolved on the 10th day of admission. His oxygen requirement progressively increased to 15 L/min of oxygen and on the 12th day of admission, he developed respiratory distress with peripheral oxygen saturation at 60% when on 15 L/min oxygen supplementation via face mask with a reservoir bag. The arterial blood gas (ABG) report revealed potential of hydrogen (pH) of 7.053, partial pressure of oxygen in artery (PaO2) of 116 millimeters of mercury (mmHg), partial pressure of carbon dioxide in artery (PaCO2) of 86.9 mmHg, and bicarbonate of 16.9 milliequivalent per liter (mEq/L). He was then shifted to the ICU.

The patient was intubated an hour later with the ventilator settings in assist control/volume control (AC/VC) mode with the respiratory rate (RR) of 15 breaths/min, tidal volume (Vt) of 350 milliliters (mL), partial end-expiratory pressure (PEEP) of 7 centimeter of water (cm H2O), and fraction of inspired oxygen (FiO2) of 100%. His peripheral oxygen saturation did not rise above 84% and the RR was recalibrated to 18 breaths/min, Vt to 360 mL, and PEEP to 9 cm H2O. The patient was then given IV sodium bicarbonate of 50 mEq bolus dose with an infusion at 10 mEq/hour. 

The patient was started on IV meropenem 1 g and IV vancomycin 1 g thrice a day as the white cell counts increased from normal range to 15000/cubic millimeter. On the 16th day of admission, he developed a fever of 100.4°F and his ABG reports showed a pH of 7.3, PaO2 of 86 mmHg, PaCO2 of 61 mmHg and bicarbonate of 31.6 mEq/L with a ventilator setting in AC/VC mode RR of 26/min, Vt of 280 mL and PEEP 10 cm H2O and FiO2 55%. His lactate was elevated to 1.19 mEq/L and he was started on IV colistin 2 million units (MU) twice a day.

**Figure 1:**
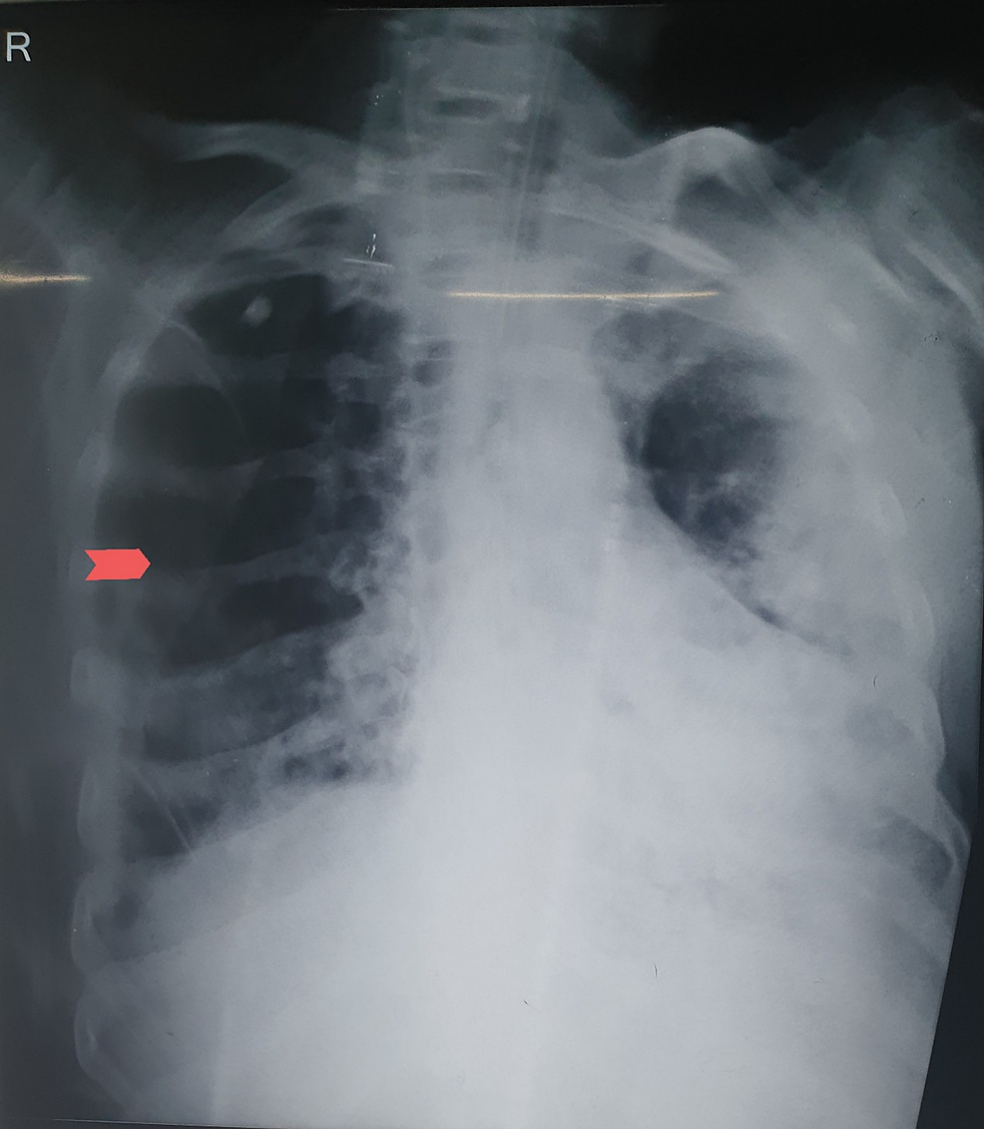
CXR demonstrating right-sided pneumothorax (shown by arrow). CXR, Chest X-ray

On the 18th day of admission, he developed spontaneous pneumothorax on the right side of the chest which was detected on routine chest X-ray (CXR) (Figure 1). The ventilator settings when he developed pneumothorax were AC/VC mode with the RR of 26/min, Vt of 280 mL, PEEP 10 cm H2O, and FiO2 55%. A right-sided chest tube was inserted to relieve the pneumothorax. On the subsequent day, his blood pressure started to drop below 70/40 mmHg, which required the initiation of IV noradrenaline infusion at the rate of 3 micrograms per kilogram per minute (mcg/kg/min). Oral metoprolol was subsequently stopped.

On the 11th day following intubation, the ventilator settings were changed to synchronized intermittent mandatory ventilation (SIMV) mode. The patient was extubated on the 12th day following intubation. However, he experienced persistent bronchospasm and respiratory distress and had to be re-intubated the same day. The routine CXR showed resolution of pneumothorax with a functioning chest drain. However, on the following day, he developed spontaneous right apical pneumothorax requiring the repositioning of the intercostal tube. The second course of IV piperacillin/tazobactam and vancomycin was started as he developed a fever. Intravenous infusion of noradrenaline was reinitiated at 0.5 mcg/kg/min. Intravenous infusion of sodium bicarbonate had to be started at the rate of 15 mEq/hour due to persistent acidosis. The central venous line (CVL) and Foley catheter were changed as well and the fever began to subside. The urine culture showed Escherichia coli only sensitive to Polymixin B and he was initiated on the same. The need for noradrenaline gradually began to decrease, but he started to develop persistent tachycardia for which injection metoprolol was started.

On the 22nd day of the first intubation, a tracheostomy was performed under general anesthesia. A vertical incision was given anteriorly on the neck, pre-tracheal fascia was incised and dissected, and a stoma was made over the second and third tracheal ring. A cuffed tube of 7.5 millimeter (mm) was placed in situ and the position was confirmed. His post-procedure peripheral oxygen saturation was 98%. 

**Figure 2:**
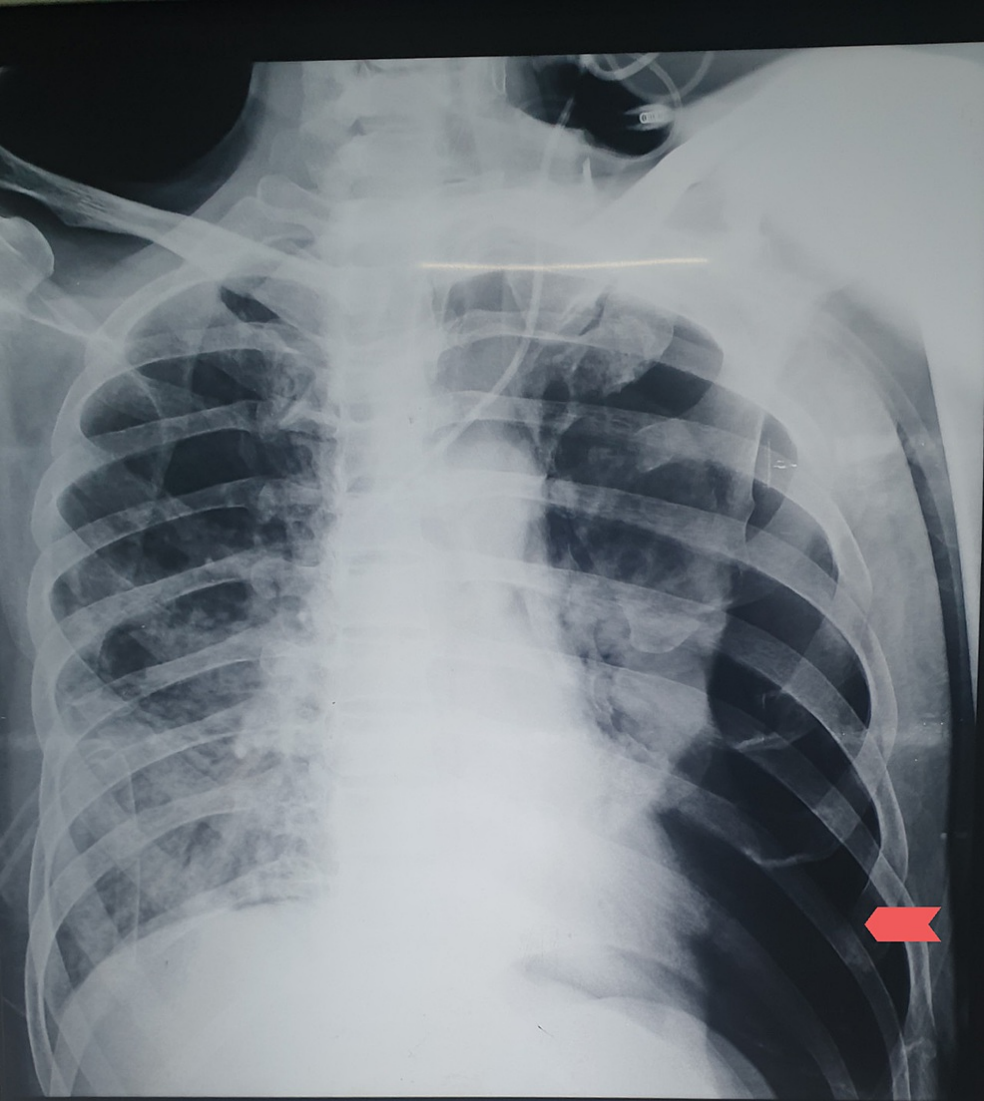
CXR demonstrating left-sided pneumothorax (shown by arrow). CXR, chest X-ray

The right-sided intercostal tube was removed on the 20th day of insertion on the basis of radiological confirmation of resolution of pneumothorax after clamping the chest drain for three hours. However, on the same day, he developed a pneumothorax on the left side of the chest (Figure 2) with ventilator in AC/VC mode with FiO2 60%, PEEP 5 cmH2O, RR of 24 breaths/ min, and Vt of 340 mL. A left-sided chest tube was inserted. Bedside ultrasonography showed minimal right-sided pleural effusion. Foley’s tip culture showed Staphylococcus species sensitive to piperacillin/tazobactam and central line culture showed Klebsiella species sensitive to cefepime and the patient was placed on these antibiotics. Based on the results of the intercostal tube culture, which showed fungal hyphae, capsule voriconazole 200 mg twice a day and IV imipenem/cilastatin were started. The white blood cell count then started to decline.

The left-sided chest tube was removed after 12 days of insertion based on the radiological and clinical resolution of pneumothorax after clamping. The intercostal drain tip culture was sent which came positive for Klebsiella sensitive to amoxicillin. The patient had intermittent episodes of fever up to 101°F on the day of the removal of the intercostal drain.

On the 43rd day following his second intubation, he was successfully extubated after a day of SIMV trial. His nutritional requirement was managed by regular dietician visits. He was doing well and was maintaining normal oxygen saturation at 2L/minute oxygen via nasal prongs or T-piece. The tracheostomy stoma was gradually narrowed by the insertion of smaller-sized cuffs.

**Figure 3:**
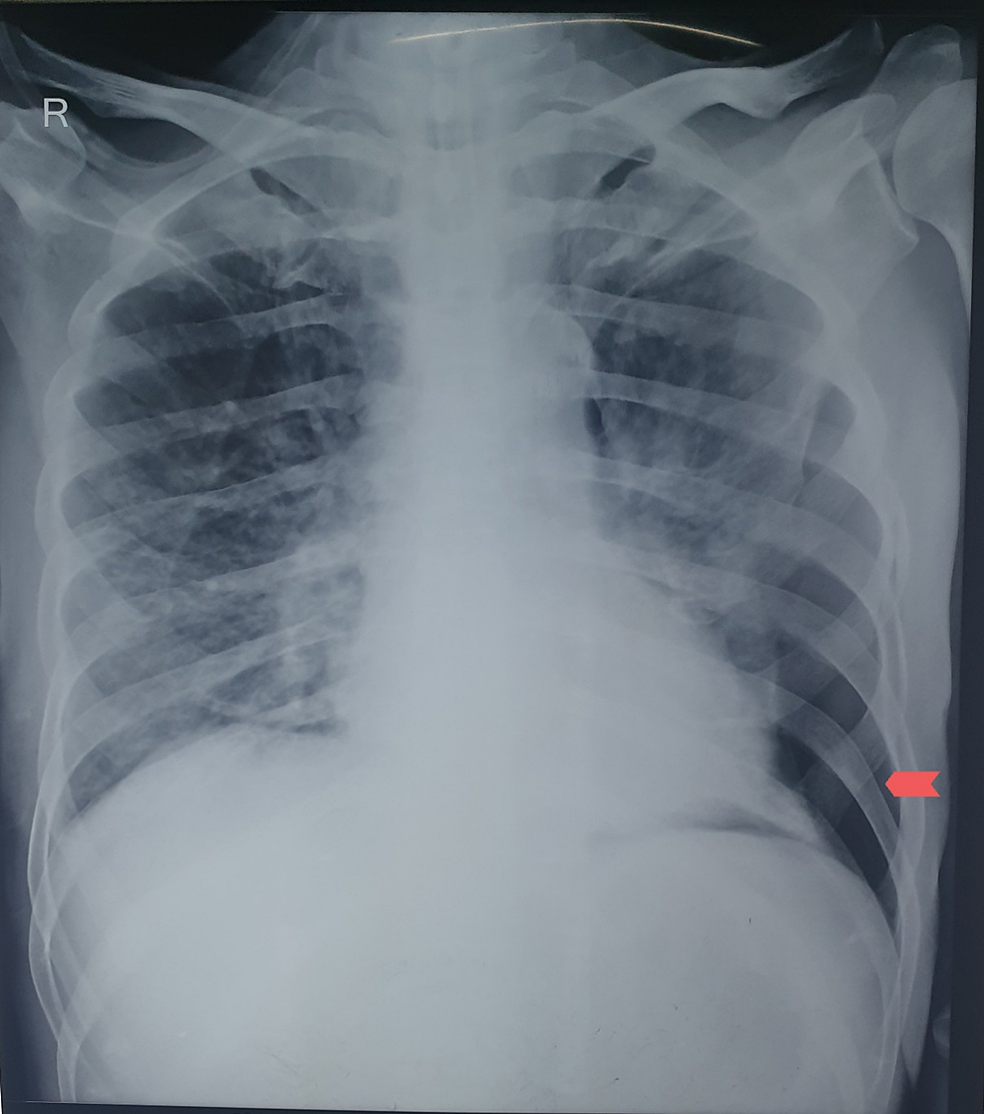
CXR demonstrating left-sided pneumothorax (shown by arrow). CXR, chest X-ray

However, on the sixth day following extubation, he developed a left-sided spontaneous pneumothorax (Figure 3) while on 2L/min oxygen via nasal prongs. A chest tube was inserted the same day. After five days of the chest tube insertion, pleurodesis with 10% povidone-iodine was performed following the fourth pneumothorax episode. 

After 37 days of tracheostomy, the tracheostomy tube was removed and the stoma closed. The left intercostal tube was removed after 10 days. At present, the patient is mobile, and under nutritional rehabilitation and chest physiotherapy. He does not require oxygen supplementation.

## Discussion

Pneumothorax is defined as the condition in which air is collected between the visceral and the parietal pleura. Common causes include males, smokers, barotrauma from ventilation, or underlying lung pathology [4]. 

Our case presented with fever and shortness of breath, which are the most common presenting symptoms of COVID-19 [5]. On imaging, patchy ground-glass opacities were seen in the peripheral region and mostly in the lower zone. Pleural effusion was also recorded in our patient in multiple chest imaging over the course of more than 80 days in the ICU. The most common imaging finding noted in patients with COVID-19 infections are ground-glass opacities which are posterior and peripheral in distribution. Uncommon imaging findings are pleural effusion, lymphadenopathy, pneumothorax, cavitation, and pericardial effusion [5]. A case of a patient with pneumomediastinum and subcutaneous pneumothorax has been reported in Nepal [6]. The current case is, to our knowledge, the first reported case of recurrent pneumothorax in association with COVID-19 in Nepal. Though causation between COVID-19 and pneumothorax cannot be established from a single case, our report adds on the literature where multiple patients with this disease have developed a pneumothorax [4, 7-13]. Spontaneous pneumothorax has also been reported as a late complication after the resolution of the infection [3]. Pneumothorax can occur in patients receiving either noninvasive or invasive modalities of ventilation likely due to the fragility of the airways weakened by the virus and barotrauma induced by oxygen supplementation devices. Pneumothorax has been commonly associated with other causes of severe acute respiratory distress syndrome caused by pressure and volume induced alveolar rupture but its causation with COVID-19 has not been established. Since our patient developed pneumothorax in both the presence and absence of ventilator support, the possible explanations are barotrauma and secondary spontaneous pneumothorax due to COVID-19 induced alveolar damage, as he had no known pulmonary pathology except COVID-19. Cyst formation could also be a possible explanation for the pneumothorax as it is a known complication in cases with severe pneumothorax. Cases of recurrent pneumothorax are rare in literature and patients with pneumothorax up to four episodes are even rarer [14]. Each episode of pneumothorax was managed with intercostal tube insertion and a closed suction bottle to avoid the spread of droplet infection. Our patient did respond to chest tube drainage following each episode. Chest tube infection was noted which was managed with IV antibiotics. After the fourth episode, to prevent further pneumothorax, pleurodesis was performed with 10% povidone and normal saline through the intercostal tube and the solution was kept in the loop in the tube for five hours.

At present, our patient is stable with peripheral oxygen saturation at 97% without supplemental oxygen. Nutritional rehabilitation and physiotherapy are being implemented for his gradual return to activities of daily living.

## Conclusions

The occurrence of multiple episodes of pneumothorax is a rare finding. A patient with COVID-19 can develop pneumothorax while on ventilator support or after extubation possibly due to barotrauma, alveolar injury, or bulla/bleb formation. There should be a high index of suspicion in any COVID-19 patients, whether in a ventilator or not, for the development of pneumothorax when they start to deteriorate. Such cases can be managed with intercostal tube insertion and in cases of recurrent pneumothorax, pleurodesis can help prevent a recurrence.

## References

[1] Parasher A (2021). COVID-19: current understanding of its pathophysiology, clinical presentation and treatment. Postgrad Med J.

[2] Mallick T, Dinesh A, Engdahl R, Sabado M (2020). COVID-19 complicated by spontaneous pneumothorax. Cureus.

[3] Nunna K, Braun AB (2021). Development of a large spontaneous pneumothorax after recovery from mild COVID-19 infection. BMJ Case Rep.

[4] Abushahin A, Degliuomini J, Aronow WS, Newman T (2020). A case of spontaneous pneumothorax 21 days after diagnosis of coronavirus disease 2019 (COVID-19) pneumonia. Am J Case Rep.

[5] Hameed M, Jamal W, Yousaf M (2020). Pneumothorax in covid-19 pneumonia: a case series. Respir Med Case Rep.

[6] Kafle S, Shrestha E, Pokharel N, Budhathoki P, Shrestha DB, Vittorio T (2021). Pneumomediastinum and subcutaneous emphysema in an adult male from Nepal infected with COVID-19. Cureus.

[7] Aiolfi A, Biraghi T, Montisci A (2020). Management of persistent pneumothorax with thoracoscopy and bleb resection in COVID-19 patients. Ann Thorac Surg.

[8] Quincho-Lopez A, Quincho-Lopez DL, Hurtado-Medina FD (2020). Case report: pneumothorax and pneumomediastinum as uncommon complications of COVID-19 pneumonia - literature review. Am J Trop Med Hyg.

[9] Eperjesiova B, Hart E, Shokr M, Sinha P, Ferguson GT (2020). Spontaneous pneumomediastinum/pneumothorax in patients with COVID-19. Cureus.

[10] Rohailla S, Ahmed N, Gough K (2020). SARS-CoV-2 infection associated with spontaneous pneumothorax. CMAJ.

[11] López Vega JM, Parra Gordo ML, Diez Tascón A, Ossaba Vélez S (2020). Pneumomediastinum and spontaneous pneumothorax as an extrapulmonary complication of COVID-19 disease. Emerg Radiol.

[12] Wang W, Gao R, Zheng Y, Jiang L (2020). COVID-19 with spontaneous pneumothorax, pneumomediastinum and subcutaneous emphysema. J Travel Med.

[13] Hollingshead C, Hanrahan J (2020). Spontaneous pneumothorax following COVID-19 pneumonia. IDCases.

[14] Martinelli AW, Ingle T, Newman J (2020). COVID-19 and pneumothorax: a multicentre retrospective case series. Eur Respir J.

